# Dyslipidemia is Associated with Unfit and Overweight-Obese Children and Adolescents

**DOI:** 10.5935/abc.20160025

**Published:** 2016-03

**Authors:** Cézane Priscila Reuter, Priscila Tatiana da Silva, Jane Dagmar Pollo Renner, Elza Daniel de Mello, Andréia Rosane de Moura Valim, Luiza Pasa, Rafaela da Silva, Miria Suzana Burgos

**Affiliations:** 1 Universidade de Santa Cruz do Sul, Santa Cruz do Sul, RS - Brazil; 2 Universidade Federal do Rio Grande do Sul, Porto Alegre, RS - Brazil

**Keywords:** Dyslipidemias, Obesity, Overweight, Physical Fitness

## Abstract

**Background:**

Both poor aerobic fitness and obesity, separately, are associated with abnormal lipid profiles.

**Objective:**

To identify possible relationships of dyslipidemia with cardiorespiratory fitness and obesity, evaluated together, in children and adolescents.

**Methods:**

This cross-sectional study included 1,243 children and adolescents (563 males and 680 females) between 7 and 17 years of age from 19 schools. Obesity was assessed using body mass index (BMI) measurements, and cardiorespiratory fitness was determined via a 9-minute run/walk test. To analyze the lipid profile of each subject, the following markers were used: total cholesterol, cholesterol fractions (high-density lipoprotein and low-density lipoprotein) and triglycerides. Data were analyzed using SPSS v. 20.0, via prevalence ratio (PR), using the Poisson regression.

**Results:**

Dyslipidemia is more prevalent among unfit/overweight-obese children and adolescents compared with fit/underweight-normal weight boys (PR: 1.25; p = 0.007) and girls (PR: 1.30, p = 0.001).

**Conclusions:**

The prevalence of dyslipidemia is directly related to both obesity and lower levels of cardiorespiratory fitness.

## Introduction

Lifestyle changes, including obesity, have increased the prevalence of dyslipidemia in both children and adolescents.^1^ Both poor aerobic fitness and poor weight management are associated with abnormal lipid profiles, and these findings emphasize that dyslipidemia screening in youth should be adopted as a new tool to promote improved public health.^2^ Additionally, both genetic and environmental factors may be determinants of dyslipidemia.^3^

In Brazil, previous studies have noted a high prevalence of lipid disorders during both childhood and adolescence. A study conducted in Campina Grande, Paraíba state, has indicated that dyslipidemia was present in 66.7% of adolescents; decreased levels of high-density lipoprotein cholesterol (HDL-C) were observed in 56.7% of these individuals.^4^ Franca and Alves^5^ have evaluated 414 healthy children and adolescents in Pernambuco state and have concluded that 29.7% exhibited undesirable lipid profiles, characterized by increased levels of triglycerides (TG), low-density lipoprotein cholesterol (LDL-C) and total cholesterol (TC).

Therefore, measuring the serum levels of TC, LDL-C, HDL-C and TG, as well as assessing other risk factors for heart disease, such as obesity and physical inactivity, is necessary to predict cardiovascular disease in the future.^6,7^ This study aims to identify possible relationships of dyslipidemia and both low levels of cardiorespiratory fitness (CRF) and obesity in both children and adolescents.

## Methods

This cross-sectional study included 1,243 children and adolescents from 19 municipal schools in Santa Cruz do Sul, Rio Grande do Sul state, Brazil, including 563 boys and 680 girls between 7 and 17 years of age (median: 12.0 years) from urban and rural areas. This study is a part of the "School Health Project", a larger longitudinal cohort that started in 2004, approved by the *Human Research Ethics Committee* of the University of Santa Cruz do Sul (UNISC), under protocol number 714.216, and conducted within the standards required by the Declaration of Helsinki. Each child's parents or guardians provided written informed consent, authorizing their child to participate in the study.

All evaluations were performed at the UNISC. Body mass index (BMI) was calculated using the following formula: BMI = weight (kg)/height (m)^2^. The values obtained were classified via the percentile curves of the CDC/NCHS^8^ and were considered underweight (< p5), normal weight (≥ p5 and < p85), overweight (p ≥ 85 and < p95) and obese (≥ p95) based on both the sex and the age of the schoolchildren. As recommended by the Sport Brazil Project,^9^ CRF was assessed via a 9-minute run/walk test. The data were classified as follows: 1) fit (high levels of CRF) and 2) unfit (low levels of CRF). Using the previously categorized BMI and CRF data, a new classification of body type was performed using these two variables together as follows: 1) fit/underweight-normal weight, 2) fit/overweight-obese, 3) unfit/underweight-normal weight and 4) unfit/overweight-obese.

Blood was collected following a 12-hour fast. The following markers were used for lipid profile analysis: TC, HDL-C, LDL-C and TG. The analyses were performed using Miura One automated equipment (ISE, Rome, Italy) and commercial kits from DiaSys (DiaSys Diagnostic Systems, Germany). LDL-C was calculated using the Friedewald equation.^10^ Thereafter, each of the values was classified according to the National Heart, Lung, and Blood Institute,^11^ which uses the following cut-off values: 1) TC: ≥ 200 mg/dL; 2) LDL-C ≥ 130 mg/dL; and 3) TG: ≥ 100 mg/dL (0-9 years) or ≥ 130 mg/dL (10-19 years). An HDL-C value < 40 mg/dL was also considered low. Schoolchildren were considered dyslipidemic if they exhibited a change in at least one of the parameters listed above.

Data analysis was performed using SPSS v. 20.0 software (IBM, Chicago, IL, USA). Descriptive analyses (numbers and percentages) were used to characterize samples. The relationships between the categorical variables, stratified by sex, were tested using the chi-square test. The relationships between dyslipidemia and both CRF and obesity in children and adolescents were tested using prevalence ratio (PR) and Poisson regression. Differences were regarded as significant if p < 0.05.

## Results

Table 1 presents the descriptive characteristics of the schoolchildren included in this study. There was a high percentage of children with dyslipidemia (42.1%) as well as of children who were either overweight or obese (29.1%) and exhibited low levels of CRF (50.8%).

**Table 1:** Sample characteristics

	**n (%)**
**Sex**	
Boys	563 (45.3)
Girls	680 (54.7)
**Total cholesterol**	
Acceptable + Borderline	902 (72.6)
High	341 (27.4)
**HDL-cholesterol**	
Acceptable + Borderline	1167 (93.9)
Low	76 (6.1)
**LDL-cholesterol**	
Acceptable + Borderline	960 (77.2)
High	283 (22.8)
**Triglycerides**	
Acceptable + Borderline	1170 (94.1)
High	73 (5.9)
**Dyslipidemia**	
No	720 (57.9)
Yes	523 (42.1)
**Body mass index (BMI)**	
Underweight + normal weight	881 (70.9)
Overweight + obesity	362 (29.1)
**Cardiorespiratory fitness**	
Fit	611 (49.2)
Unfit	632 (50.8)
**Cardiorespiratory fitness/BMI**	
Fit/Underweight-normal weight	479 (38.5)
Fit/Overweight-obesity	132 (10.7)
Unfit/Underweight-normal weight	402 (32.3)
Unfit/Overweight-obesity	230 (18.5)

The data in Table 2 indicate that dyslipidemia was more prevalent among girls (p < 0.001). Additionally, a higher percentage of fit/underweight-normal weight (40.3%) subjects was noted among the boys compared with the girls (37.1%).

**Table 2:** Dyslipidemia and combined cardiorespiratory fitness (CRF) and body mass index (BMI) by sex

	**Boys (n = 563)**	**Girls (n = 680)**	p*
	**n (%)**	**n (%)**	
Dyslipidemia			
No	357 (63.4)	363 (53.4)	
Yes	206 (36.6)	317 (46.6)	< 0.001
CRF/BMI			
Fit/Underweight-normal weight	227 (40.3)	252 (37.1)	
Fit/Overweight-obesity	77 (13.7)	55 (8.1)	
Unfit/Underweight-normal weight	150 (26.6)	252 (37.1)	< 0.001
Unfit/Overweight-obesity	109 (19.4)	121 (17.7)	

* Chi-square test

Table 3 depicts the relationship between dyslipidemia and CRF/BMI. Following an adjustment for age, dyslipidemia was more prevalent among the unfit/overweight-obese schoolchildren compared with the fit/underweight-normal weight schoolchildren, for both boys (PR: 1.25; p = 0.007) and girls (PR: 1.30; p = 0.001).

**Table 3:** The relationship among dyslipidemia and combined cardiorespiratory fitness and body mass index

	**Dyslipidemia**	**p**	**Dyslipidemia**	**p**
	**Crude PR (95% CI)**		Adjusted PR* (95% CI)	
**Boys**				
Fit/Underweight-normal weight	1		1	
Fit/Overweight-obesity	1.05 (0.96-1.15)	0.317	1.20 (0.99-1.44)	0.062
Unfit/Underweight-normal weight	0.97 (0.90-1.04)	0.424	0.80 (0.71-0.92)	0.001
Unfit/Overweight-obesity	1.10 (1.03-1.20)	0.009	1.25 (1.06-1.46)	0.007
**Girls**				
Fit/Underweight-normal weight Fit/Overweight-obesity	1 0.99 (0.89-1.10)	0.846	1 1.12 (0.90-1.39)	0.317
Unfit/Underweight-normal weight	1.01 (0.95-1.07)	0.857	0.91 (0.81-1.02)	0.091
Unfit/Overweight-obesity	1.13 (1.05-1.21)	0.001	1.30 (1.11-1.51)	0.001

Poisson regression; PR: prevalence ratio; CI: confidence interval;

* for age

We analyzed the lipid profiles separately. As shown in Table 4, high levels of TG were more prevalent among the unfit/overweight-obese subjects for both boys (PR: 1.08; p = 0.017) and girls (PR: 1.11; p = 0.001). High TC was associated only with an unfit/overweight-obese body type among boys (PR: 1.09; p = 0.036). Table 4 also indicates that there were no significant differences between HDL-C and LDL-C where CRF and BMI were concerned.

**Table 4:** The relationship between high levels of TG, TC, HDL-C and LDL-C and combined CRF and BMI

	**TG**	**p**	**TC**	**p**
	Adjusted PR* (95%CI)		Adjusted PR* (95%CI)	
**Boys**				
Fit/Underweight-normal weight	1		1	
Fit/Overweight-obesity	1.03 (0.97-1.09)	0.310	1.02 (0.93-1.11)	0.721
Unfit/Underweight-normal weight	0.99 (0.96-1.01)	0.258	1.02 (0.95-1.09)	0.613
Unfit/Overweight-obesity	1.08 (1.01-1.14)	0.017	1.09 (1.01-1.18)	0.036
**Girls**				
Fit/Underweight-normal weight	1		1	
Fit/Overweight-obesity	1.11 (1.02-1.20)	0.020	0.99 (0.89-1.01)	0.989
Unfit/Underweight-normal weight	1.01 (0.98-1.05)	0.470	1.01 (0.95-1.08)	0.659
Unfit/Overweight-obesity	1.11 (1.05-1.18)	0.001	1.06 (0.98-1.15)	0.132
	**HDL-C**	**p**	**LDL-C**	**p**
	Adjusted PR* (95%CI)		Adjusted PR* (95%CI)	
**Boys**				
Fit/Underweight-normal weight	1		1	
Fit/Overweight-obesity	1.02 (0.96-1.09)	0.510	1.05 (0.96-1.15)	0.309
Unfit/Underweight-normal weight	1.01 (0.97-1.06)	0.557	0.97 (0.91-1.04)	0.362
Unfit/Overweight-obesity	1.05 (0.99-1.12)	0.084	1.00 (0.93-1.08)	0.920
**Girls**				
Fit/Underweight-normal weight	1		1	
Fit/Overweight-obesity	1.00 (0.94-1.06)	0.961	0.94 (0.85-1.04)	0.216
Unfit/Underweight-normal weight	0.99 (0.96-1.03)	0.719	0.94 (0.89-1.00)	0.057
Unfit/Overweight-obesity	1.03 (0.97-1.08)	0.357	1.04 (0.96-1.12)	0.314

Poisson regression; PR: prevalence ratio; CI: confidence interval; TG: triglycerides; TC: total cholesterol; HDL-C: high-density lipoprotein cholesterol; LDL-C: low-density lipoprotein cholesterol;

* for age

## Discussion

In this study, dyslipidemia was more prevalent among girls (46.6%) compared with boys (36.6%). The results are similar to those observed among children in Norway, where females exhibited less favorable lipid profiles characterized by higher TG levels (p = 0.007) and LDL-C levels (p = 0.013) and lower concentrations of HDL-C (p = 0.004) compared with boys.^12^ In Brazil, a study conducted with preschoolers of the city of Diamantina, Minas Gerais state, has shown that the prevalence of dyslipidemia was 65.19%.^13^

Recent studies have found that children at risk for becoming overweight who had high levels of CRF had superior metabolic profiles compared with children who were at risk for becoming overweight, who exhibited low levels of CRF. High levels of CRF may reduce the impact of BMI on the development of metabolic syndrome and result in improved metabolic profiles,^14-16^ suggesting that CRF reduces the overall cardiometabolic risk among children.^17^

The data from this study indicate that increased body fat, as well as CRF, seems to influence the occurrence of dyslipidemia. When adjusted for age, dyslipidemia was more prevalent among unfit/overweight-obese schoolchildren, compared with fit/underweight-normal weight schoolchildren for both boys (PR: 1.25; p = 0.007) and girls (PR: 1.30; p = 0.001). Additionally, both boys and girls within the fit/underweight-normal weight group exhibited a lower prevalence of dyslipidemia (PR: 0.80 and 0.91, respectively) compared with their fit/overweight-obese counterparts (PR: 1.20 and 1.12, respectively), suggesting that adequate BMI may be a more important health factor than higher levels of CRF among schoolchildren.

A similar result has been observed in a study conducted in Athena, Greece, which included 2,410 children and examined the differences in cardiometabolic risk factors among children with different BMI profiles and CRF levels. The results of the study indicated that the groups of children that were classified as "leaner and less fit" exhibited lower levels of TG and TC and increased levels of HDL-C compared with "heavier and more fit" children.^18^ Another study conducted in China that included 676 students has observed that children with lower levels of fat have a lower risk of developing metabolic syndrome.^16^

Telford et al.^19^ have observed that blood lipids are sensitive to normal changes that occur in both body fat percentage and CRF among adolescents, suggesting that attention should be paid to body composition to prevent the development of cardiovascular disease during adulthood. In young adults, Vranian et al.^20^ found that both obesity and CRF are associated with an increased cardiometabolic risk and that their effects are cumulative. However, obesity is more strongly associated with metabolic disorders, highlighting the importance of the combination of weight loss and improved CRF. In Spain, BMI mediates the relationship between CRF and metabolic syndrome among schoolchildren, emphasizing that high levels of CRF are associated with a lower cardiometabolic risk, particularly in the setting of weight loss.^21^ In the city of Vitória, Espírito Santo state, Brazil, a study with adolescents has revealed that low levels of CRF are negatively related to cardiovascular risk factors, particularly overweight.^22^

Therefore, knowing that changes in lipid profile can cause problems for children's health, such as atherosclerosis, early prevention, by adopting and maintaining a healthy lifestyle, is fundamental.^23^

Our study reinforces the important relationship between dyslipidemia and being overweight or obese and having low levels of CRF among both children and adolescents. In addition, we concurrently evaluated CRF and obesity in a representative sample. To our knowledge, the associations we examined have not been previously evaluated for Brazilian children and adolescents. Associations of both poor aerobic fitness and obesity with abnormal lipid profiles have only been separately demonstrated in prior studies. However, our study had some limitations. Although the 9-minute run/walk test is widely used in Brazil, it does not include a formula for predicting VO_2_ max. Additionally, this study employed a cross-sectional design that did not follow the schoolchildren over time.

## Conclusion

The present study demonstrated that dyslipidemia is more prevalent among unfit/overweight and obese children and adolescents than among their fit/underweight-normal weight counterparts. Therefore, this investigation suggests that therapeutic interventions and interdisciplinary practices are important for both obesity prevention and control, as are encouraging physical activity and avoiding future health problems.
